# Gauge fixing for sequence-function relationships

**DOI:** 10.1371/journal.pcbi.1012818

**Published:** 2025-03-20

**Authors:** Anna Posfai, Juannan Zhou, David M. McCandlish, Justin B. Kinney

**Affiliations:** 1 Simons Center for Quantitative Biology, Cold Spring Harbor Laboratory, Cold Spring Harbor, New York, United States of America; 2 Department of Biology, University of Florida, Gainesville, Florida, United States of America; University of Cambridge, UNITED KINGDOM OF GREAT BRITAIN AND NORTHERN IRELAND

## Abstract

Quantitative models of sequence-function relationships are ubiquitous in computational biology, e.g., for modeling the DNA binding of transcription factors or the fitness landscapes of proteins. Interpreting these models, however, is complicated by the fact that the values of model parameters can often be changed without affecting model predictions. Before the values of model parameters can be meaningfully interpreted, one must remove these degrees of freedom (called “gauge freedoms” in physics) by imposing additional constraints (a process called “fixing the gauge”). However, strategies for fixing the gauge of sequence-function relationships have received little attention. Here we derive an analytically tractable family of gauges for a large class of sequence-function relationships. These gauges are derived in the context of models with all-order interactions, but an important subset of these gauges can be applied to diverse types of models, including additive models, pairwise-interaction models, and models with higher-order interactions. Many commonly used gauges are special cases of gauges within this family. We demonstrate the utility of this family of gauges by showing how different choices of gauge can be used both to explore complex activity landscapes and to reveal simplified models that are approximately correct within localized regions of sequence space. The results provide practical gauge-fixing strategies and demonstrate the utility of gauge-fixing for model exploration and interpretation.

## Author summary

Biophysics and other areas of quantitative biology rely heavily on mathematical models that predict biological activities from DNA, RNA, or protein sequences. Interpreting the parameters of these models, however, is not trivial. Here we address a core challenge for model interpretation–the presence of “gauge freedoms”, i.e., directions in parameter space that do not affect model predictions and therefore cannot be constrained by data. Our results provide an explicit mathematical method for removing these unconstrained degrees of freedom–a process called “fixing the gauge”–that can be applied to a wide range of commonly use models of sequence-function relationships, including models that describe interactions of arbitrarily high order. These results unify diverse gauge fixing methods that have been previously described in the literature for specific types of models. We further show how our gauge-fixing approach can be used to simplify complex models in user-specified regions of sequence space. This work thus overcomes a major obstacle in the interpretation of quantitative sequence-function relationships.

## Introduction

One of the central challenges of biology is to understand how functionally relevant information is encoded within DNA, RNA, and protein sequences. Unlike the genetic code, most sequence-function relationships are quantitative in nature, and understanding them requires finding mathematical functions that, upon being fed unannotated sequences, return values that quantify sequence activity [1]. Multiplex assays of variant effects (MAVEs), functional genomics methods, and other high-throughput techniques are rapidly increasing the ease with which sequence-function relationships can be experimentally studied. And while quantitative modeling efforts based on these high-throughput data are becoming increasingly successful, in that they yield models with ever-increasing predictive ability, major open questions remain about how to interpret both the parameters [2–12] and the predictions [13–17] of the resulting models. One major open question is how to deal with the presence of gauge freedoms.

Gauge freedoms are directions in parameter space along which changes in model parameters have no effect on model predictions [18]. Not only can the values of model parameters along gauge freedoms not be determined from data, differences in parameters along gauge freedoms have no biological meaning even in principle. Many commonly used models of sequence-function relationships exhibit numerous gauge freedoms [19–35], and interpreting the parameters of these models requires imposing additional constraints on parameter values, a process called “fixing the gauge”.

The gauge freedoms of sequence-function relationships are most completely understood in the context of additive models (commonly used to describe transcription factor binding to DNA [19,22,35]) and pairwise-interaction models (commonly used to describe proteins [20,21,23–34]). Recently, some gauge-fixing strategies have been described for all-order interaction models, again in the context of protein sequence-function relationships [30,31,34]. However, a unified gauge-fixing strategy applicable to diverse models of sequence-function relationships has yet to be developed.

Here we provide a general treatment of the gauge fixing problem for sequence-function relationships, focusing on the important case where the set of gauge-fixed parameters form a vector space. These “linear gauges” predominate in the literature (though there are exceptions [36,37]), and have the useful property that differences between vectors of gauge-fixed parameter values are directly interpretable. We first demonstrate the relationship between these linear gauges and $L_{2}$ regularization on parameter vectors, and then derive a mathematically tractable family of gauges for the all-order interaction model. Importantly, a subset of these gauges–the “hierarchical gauges”–can be applied to diverse models beyond just the all-order interaction model (including additive models, pairwise-interaction models, and higher-order interaction models) and include as special cases two types of gauges that are commonly used in practice (“zero-sum gauges” [23,28] and “wild-type gauges” [9,23,33]). We then illustrate the properties of this family of gauges by analyzing two example sequence-function relationships: a simulated all-order interaction landscape on short binary sequences, and an empirical pairwise-interaction landscape for the B1 domain of protein G (GB1). The GB1 analysis, in particular, shows how different hierarchical gauges can be used to explore, simplify, and interpret complex functional landscapes. A companion paper [38] further explores the mathematical origins of gauge freedoms in models of sequence-function relationships, and shows how gauge freedoms arise as a consequence of the symmetries of sequence space.

## Results

### Preliminaries and background

In this section we review how gauge freedoms arise in commonly used models of sequence-function relationships, as well as strategies commonly used to fix the gauge. In doing so, we establish notation and concepts that are used in subsequent sections.

#### Linear models.

We define quantitative models of sequence-function relationships as follows. Let *A* denote an alphabet comprising *α* distinct characters (written $c_{1},\ldots ,c_{\alpha }$), let *S* denote the set of sequences of length *L* built from these characters, and let $N=\alpha^{L}$ denote the number of sequences in *S*. A quantitative model of a sequence-function relationship (henceforth “model”) is a function $f(s;\vec{\theta })$ that maps each sequence *s* in *S* to a real number. The vector $\vec{\theta }$ represents the parameters on which this function depends and is assumed to comprise *M* real numbers. $s_{l}$ denotes the character at position *l* of sequence *s*. We use *l*, $l^{\prime }$, etc. to index positions (ranging from 1 to *L*) in a sequence and *c*, $c^{\prime }$, etc. to index characters in *A*.

A linear model is a model that is a linear function of $\vec{\theta }$. Linear models have the form


$$\begin{array}{ll} f(s;\vec{\theta })=\vec{\theta }\cdot \vec{x}(s)=\overset{M}{\underset{i=1}{\sum }}\theta_{i}x_{i}(s), & \; \end{array}$$ (1)

where $\vec{x}(\cdot )$ is a vector of *M* distinct sequence features and each sequence feature $x_{i}(\cdot )$ is a function that maps sequences to the real numbers. We refer to the space ${\mathbb{R}}^{M}$ in which $\vec{x}(\cdot )$ lives as feature space, and the specific vector $\vec{x}(s)$ as the embedding of sequence *s* in feature space. We use *E* to denote the vector space spanned by the set of embeddings $\vec{x}(s)$ for all sequences *s* in *S*. We emphasize that *E* is often a proper subspace of ${\mathbb{R}}^{M}$ (i.e., has dimension less than *M*). Indeed, this is what causes *f* to have gauge freedoms.

#### One-hot models.

One-hot models are linear models based on sequence features that indicate the presence or absence of specific characters at specific positions within a sequence [1]. Such models play a central role in scientific reasoning concerning sequence-function relationships because their parameters can be interpreted as quantitative contributions to the measured function due to the presence of specific biochemical entities (e.g. nucleotides or amino acids) at specific positions in the sequence. These one-hot models include additive models, pairwise-interaction models, all-order interaction models, and more. Additive models have the form


$$\begin{array}{ll} f_{add}(s)=\theta_{0}x_{0}(s)+\underset{l}{\sum }\underset{c}{\sum }\theta_{l}^{c}x_{l}^{c}(s), & \; \end{array}$$ (2)

where $x_{0}(s)$ is the constant feature (equal to one for every sequence *s*) and $x_{l}^{c}(s)$ is an additive feature (equal to one if sequence *s* has character *c* at position *l* and equal to zero otherwise; note that *c* is used here as a superscript and not a power). Pairwise interaction models have the form


$$\begin{array}{ll} f_{pair}(s)=\theta_{0}x_{0}(s)+\underset{l}{\sum }\underset{c}{\sum }\theta_{l}^{c}x_{l}^{c}(s)+\underset{l<l^{\prime }}{\sum }\underset{c,c^{\prime }}{\sum }\theta_{ll^{\prime }}^{cc^{\prime }}x_{ll^{\prime }}^{cc^{\prime }}(s), & \; \end{array}$$ (3)

where $x_{ll^{\prime }}^{cc^{\prime }}(s)$ is a pairwise feature (equal to one if *s* has character *c* at position *l* and character $c^{\prime }$ at position $l^{\prime }$, and equal to zero otherwise). All-order interaction models include interactions of all orders and have the form


$$\begin{array}{ll} f_{all}(s)=\overset{L}{\underset{K=0}{\sum }}\underset{l_{1}<\ldots <l_{K}}{\sum }\underset{c_{1},\ldots ,c_{K}}{\sum }\theta_{l_{1}\ldots l_{K}}^{c_{1}\ldots c_{K}}x_{l_{1}\ldots l_{K}}^{c_{1}\ldots c_{K}}(s), & \; \end{array}$$ (4)

where $x_{l_{1}l_{2}\ldots l_{K}}^{c_{1}c_{2}\ldots c_{K}}(s)$ is a *K*-order feature (equal to one if *s* has character $c_{k}$ at position $l_{k}$ for all *k*, and equal to zero otherwise; *K* = 0 corresponds to the constant feature).

#### Gauge freedoms.

Gauge freedoms are transformations of model parameters that leave all model predictions (i.e., the values *f* (*s*) at all sequences *s*) unchanged. The gauge freedoms of a general sequence-function relationship *f*(⋅, ⋅) are vectors $\vec{g}$ in ${\mathbb{R}}^{M}$ that satisfy


$$\begin{array}{ll} f(s;\vec{\theta })=f(s;\vec{\theta }+\vec{g})\text{ for all }s\in S. & \; \end{array}$$ (5)

For linear models, gauge freedoms $\vec{g}$ satisfy


$$\begin{array}{ll} X\vec{g}=\vec{0}, & \; \end{array}$$ (6)

where *X* is the *N* × *M* design matrix having rows $\vec{x}(s)$ for *s* ∈ *S*. In linear models, gauge freedoms thus arise when sequence features (i.e., the columns of *X*) are not linearly independent. In such cases, the space *E* spanned by sequence embeddings is a proper subspace of ${\mathbb{R}}^{M}$, the space *G* of gauge freedoms is also a proper subspace, and *G* is orthogonal to *E*.

**Fig 1:**
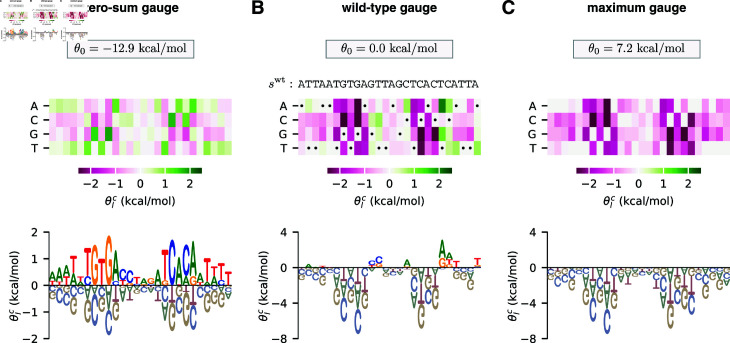
Choice of gauge impacts model parameters. (A–C) Parameters, expressed in three different gauges, for an additive model describing the (negative) binding energy of the *E. coli* transcription factor CRP to DNA. Model parameters are from [37]. In each panel, additive parameters $\theta_{l}^{c}$ are shown using both (top) a heat map and (bottom) a sequence logo [39]. The value of the constant parameter $\theta_{0}$ is also shown. (A) The zero-sum gauge, in which the additive parameters at each position sum to zero. (B) The wild-type gauge, in which the additive parameters at each position quantify activity differences with respect to a wild-type sequence, $s^{\text{wt}}$. The wild-type sequence used here (indicated by dots on the heat map) is the CRP binding site present at the *E. coli* lac promoter. (C) The maximum gauge, in which the additive parameters at each position quantify differences with respect to the optimal character at that position. Note that, while the value of each additive parameter $\theta_{l}^{c}$ varies between panels A-C, differences of the form $\theta_{l}^{c}–\theta_{l}^{c^{\prime }}$ are preserved.

Each linear relation between multiple columns of *X* yields a gauge freedom. For example, additive models have *L* gauge freedoms arising from the *L* linear relations,


$$\begin{array}{ll} x_{0}(s)=\underset{c}{\sum }x_{l}^{c}(s), & \; \end{array}$$ (7)

for all positions *l*. Pairwise models have *L* gauge freedoms arising from the *L* additive model linear relations in Eq (7), and L2(2α − 1) additional gauge freedoms arising from the linear relations


$$\begin{array}{ll} x_{l}^{c}(s)=\underset{c^{\prime }}{\sum }x_{ll^{\prime }}^{cc^{\prime }}(s)\text{ and }x_{l^{\prime }}^{c^{\prime }}(s)=\underset{c}{\sum }x_{ll^{\prime }}^{cc^{\prime }}(s) & \; \end{array}$$ (8)

for all characters $c,c^{\prime }$ and all positions *l* and $l^{\prime }$, with $l<l^{\prime }$ (see 2 for details). More generally, the gauge freedoms of one-hot models arise from the fact that summing any *K*-order feature $x_{l_{1}\ldots l_{K}}^{c_{1}\ldots c_{K}}$ over all characters $c_{k}$ at any chosen position $l_{k}$ yields a feature of order *K*–1. A proof that all gauge freedoms arise from such constraints is given in our companion paper [38].

#### Parameter values depend on choice of gauge.

Gauge freedoms pose problems for the interpretation of model parameters (e.g., when interpreting attribution maps from genomic AI models [40]) because, when gauge freedoms are present, different choices of model parameters can give the exact same model predictions. Thus, unless constraints are placed on the values of allowable parameters, individual parameters will have little biological meaning when viewed in isolation. To interpret model parameters, one therefore needs to adopt constraints that eliminate gauge freedoms and, as a result, make the values of model parameters unique. Geometrically, this means restricting model parameters to a subspace *Θ*, called “the gauge”, on which these constraints are satisfied. This process of choosing constraints (i.e., choosing *Θ*) is called “fixing the gauge”. There are many different gauge-fixing strategies. For example, Fig 1 shows an additive model of the DNA binding energy of CRP (an important transcription factor in *Escherichia coli* [41]) expressed in three different choices of gauge.

Fig 1A shows parameters expressed in the “zero-sum gauge” [23,28] (also called the “Ising gauge” [28], or the “hierarchical gauge” [9]). In the zero-sum gauge, the constant parameter is the mean sequence activity and the additive parameters quantify deviations from this mean activity. The name of the gauge comes from the fact that the additive parameters at each position sum to zero. The zero-sum gauge is commonly used in additive models of protein-DNA binding [35,42–47]. As we will see, zero-sum gauges are readily defined for models with pairwise and higher-order interactions as well.

**Fig 2:**
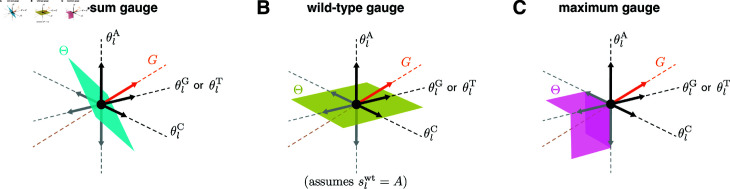
Geometry of gauge spaces for additive one-hot models. (A–C) Geometric representation of the gauge space *Θ* to which the additive parameters at each position *l* are restricted in the corresponding panel of Fig 1. Each of the four sequence features ($\theta_{l}^{A}$, $\theta_{l}^{C}$, $\theta_{l}^{G}$, and $\theta_{l}^{T}$) corresponds to a different axis. Note that the two axes for $\theta_{l}^{G}$ and $\theta_{l}^{T}$ are shown as one axis to enable 3D visualization. Black and gray arrows respectively denote unit vectors pointing in the positive and negative directions along each axis. *G* indicates the space of gauge transformations.

Fig 1B shows parameters expressed in the “wild-type gauge” [9,23,33] (also called the “lattice-gas gauge” [28] or the “mismatch gauge” [35]). In the wild-type gauge, the constant parameter is equal to the activity of a chosen wild-type sequence (denoted $s^{\text{wt}}$), and additive parameters are the changes in activity that result from mutations away from the wild-type sequence. The wild-type gauge is commonly used to visualize the results of mutational scanning experiments on proteins [48–52] or on long DNA regulatory sequences [53–58]. As we will see, wild-type gauges are also readily defined for models with pairwise and higher-order interactions.

Fig 1C shows parameters expressed in what we call the “maximum gauge”. In the maximum gauge, the constant parameter is equal to the activity of the highest-activity sequence, and additive parameters are the changes in activity that result from mutations away from this sequence. The maximum gauge is less common in the literature than the zero-sum gauge or wild-type gauge, but has been used in multiple publications [36,37].

#### Linear gauges.

Here and throughout the rest of this paper we focus on linear gauges, i.e., choices of *Θ* that are linear subspaces of feature space. For example, the zero-sum gauge and wild-type gauge (Fig 2A and 2B) are two commonly used linear gauges, whereas the maximum gauge (Fig 2C) is not a linear gauge. Linear gauges are the most mathematically tractable family of gauges. Linear gauges also have the attractive property that the difference between any two parameter vectors in *Θ* is also in *Θ*. This property makes the comparison of models within the same gauge straight-forward.

Parameters can be fixed to any chosen linear gauge via a corresponding linear projection. Formally, for any linear gauge *Θ* there exists an *M* × *M* projection matrix *P* that projects any vector ${\vec{\theta }}_{init}$ along the gauge space *G* to an equivalent vector ${\vec{\theta }}_{\text{fixed}}$ that lies in *Θ*, i.e.


$$\begin{array}{ll} {\vec{\theta }}_{\text{fixed}}=P\;{\vec{\theta }}_{init}. & \; \end{array}$$ (9)

See S1 Text Sec 3 for a proof. We emphasize that *P* depends on the choice of *Θ*, and that *P* is an orthogonal projection only for the specific choice *Θ* = *E*.

Parameters can also be gauge-fixed through a process of constrained optimization. Let *Λ* be any positive-definite *M* × *M* matrix, and let $\vec{y}=X{\vec{\theta }}_{init}$ be the *N*-dimensional vector of model predictions on all sequences. Then *Λ* specifies a unique gauge-fixed set of parameters that preserves $\vec{y}$ via


$$\begin{array}{ll} {\vec{\theta }}_{\mathrm{fi}xed}=\underset{\vec{\theta }\;:\;X\vec{\theta }=\vec{y}}{argmin}\;∥\vec{\theta }{∥}_{\Lambda^{2}},\text{ where }∥\vec{\theta }{∥}_{\Lambda^{2}}={\vec{\theta }}^{⊤}\Lambda \vec{\theta }. & \; \end{array}$$ (10)

We call *Λ* the “penalization matrix” because it determines how much each direction in parameter space is penalized in Eq (10). The resulting gauge space comprises the set of vectors that minimize the *Λ*-norm $∥\vec{\theta }{∥}_{\Lambda }$ in each gauge orbit, where the gauge orbit of a parameter vector $\vec{\theta }$ is the set of equivalent vectors $\vec{\theta }\;+\;\vec{g}$ for all *g* ∈ *G*. The corresponding projection matrix is


$$\begin{array}{ll} P=\Lambda^{–1/2}{\left(X\Lambda^{–1/2}\right)}^{+}X, & \; \end{array}$$ (11)

where ‘+’ indicates the Moore-Penrose pseudoinverse. See S1 Text Sec 3 for a proof. In what follows, the connection between the penalization matrix *Λ* and the projection matrix *P* will be used to help interpret the constraints imposed by the gauge space *Θ*.

One consequence of Eq (10) is that parameter inference carried out using a positive-definite $L_{2}$ regularizer *Λ* on model parameters will result in gauge-fixed model parameters in the specific linear gauge determined by *Λ* (see S1 Text Sec 3). While it might then seem that $L_{2}$ regularization on parameter values during inference solves the gauge fixing problem, it is important to understand that such regularization will also change model predictions (i.e., the value of *f*), whereas gauge-fixing itself influences only the values of parameters while keeping the model predictions fixed. In addition, we show in S1 Text Sec 3 that, for any desired positive-definite regularizer on model predictions and choice of linear gauge *Θ*, we can construct a penalization matrix *Λ* that imposes the desired regularization on model predictions and yields inferred parameters in the desired gauge. Thus while $L_{2}$ regularization during parameter inference can simultaneously fix the gauge and regularize model predictions, the regularization imposed on model predictions does not constrain the choice of gauge.

### Unified approach to gauge fixing

We now derive strategies for fixing the gauge of the all-order interaction model. We first introduce a geometric formulation of the all-order interaction model embedding. We then construct a parametric family of gauges for the all-order interaction model, and derive formulae for the corresponding projection and penalization matrices. Next, we highlight specific gauges of interest in this parametric family. We focus in particular on the “hierarchical gauges”, which can be applied to a variety of commonly used models in addition to the all-order interaction model. The results provide explicit gauge-fixing formulae that can be applied to diverse quantitative models of sequence-function relationships.

#### All-order interaction models.

To aid in our discussion of the all-order interaction model [Eq (4)], we define an augmented alphabet $A^{\prime }=\{*,c_{1},\ldots ,c_{\alpha }\}$, where $c_{1},\ldots ,c_{\alpha }$ are the characters in *A* and ∗ is a wild-card character that is interpreted as matching any character in *A*. Let $S^{\prime }$ denote the set of sequences of length *L* comprising characters from $A^{\prime }$. For each augmented sequence $s^{\prime }\in S^{\prime }$, we define the sequence feature $x_{s^{\prime }}(s)$ to be 1 if a sequence *s* matches the pattern described by $s^{\prime }$ and to be 0 otherwise. In this way, each augmented sequence $s^{\prime }$ serves as a regular expression against which bona fide sequences are compared.

Assigning one parameter $\theta_{s^{\prime }}$ to each of the *M* = (*α* + $1{)}^{L}$ augmented sequences $s^{\prime }$, the all-order interaction model can be expressed compactly as


$$\begin{array}{ll} f_{\text{all}}(s;\vec{\theta })=\underset{s^{\prime }\in S^{\prime }}{\sum }\theta_{s^{\prime }}x_{s^{\prime }}(s). & \; \end{array}$$ (12)

In this notation, the constant parameter $\theta_{0}$ is written $\theta_{*\cdots *}$, each additive parameter $\theta_{l}^{c}$ is written $\theta_{*\cdots c\cdots *}$, each pairwise-interaction parameter $\theta_{ll^{\prime }}^{cc^{\prime }}$ is written $\theta_{*\cdots c\cdots c^{\prime }\cdots *}$, and so on. (Here *c* occurs at position *l*, $c^{\prime }$ occurs at position $l^{\prime }$, and ⋯ denotes a run of ∗ characters). We thus see that augmented sequences provide a convenient way to index the features and parameters of the all-order interaction model.

Next we observe that $x_{s^{\prime }}$ can be expressed in a form that factorizes across positions. For each position *l*, we define $x_{l}^{*}(s)=1$ for all sequences *s* and take $x_{l}^{c_{1}},\ldots ,x_{l}^{c_{\alpha }}$ to be the standard one-hot sequence features. $x_{s^{\prime }}$ can then be written in the factorized form,


$$\begin{array}{ll} x_{s^{\prime }}(s)=\overset{L}{\underset{l=1}{\prod }}x_{l}^{s_{l}^{\prime }}(s). & \; \end{array}$$ (13)

From this it is seen that the embedding for the all-order interaction model, ${\vec{x}}_{\text{all}}(s)$, can be formulated geometrically as a tensor product:


$$\begin{array}{ll} {\vec{x}}_{\text{all}}(s)=\overset{L}{\underset{l=1}{⊗}}{\vec{x}}_{l}^{\prime }(s),\text{ where }{\vec{x}}_{l}^{\prime }(s)=\left(\;\;\begin{array}{c} x_{l}^{*}(s)\\ x_{l}^{c_{1}}(s)\\ \vdots \\ x_{l}^{c_{\alpha }}(s) \end{array}\;\;\right). & \; \end{array}$$ (14)

See S1 Text Sec 4 for details.

#### Parametric family of gauges.

We now define a useful parametric family of gauges for the all-order interaction model. As we will show, this family includes all of the most commonly used gauges in the literature (but not some less commonly used gauges, e.g., the maximum gauge [36,37]). Each gauge in this family is defined by two parameters, *λ* and *p*. *λ* is a non-negative real number that governs how much higher-order versus lower-order sequence features are penalized [in the sense of Eq (10)]. *p* is a probability distribution on sequence space that governs how strongly the specific characters at each position are penalized. This distribution is assumed to have the form


$$\begin{array}{ll} p(s)=p_{1}^{s_{1}}p_{2}^{s_{2}}\cdots p_{L}^{s_{L}}, & \; \end{array}$$ (15)

where $p_{l}^{c}$ denotes the probability of character *c* at position *l*. This assumption excludes distributions that have correlations between positions. But as we show below, choosing appropriate values for *λ* and *p* nevertheless recovers the most commonly used linear gauges, including the zero-sum gauge, the wild-type gauge, and more.

Gauges in the parametric family have analytically tractable projection matrices because each gauge can be expressed as a tensor product of single-position gauge spaces. Let $\Theta_{l}^{\lambda ,p}$ be the *α*-dimensional subspace of ${\mathbb{R}}^{\alpha +1}$ defined by


$$\begin{array}{ll} \Theta_{l}^{\lambda ,p}=V_{\lambda }⊕V_{⊥}^{p_{l}}, & \; \end{array}$$ (16)

where $V_{\lambda }$ (a 1-dimensional subspace) and $V_{⊥}^{p_{l}}$ [an (*α* *–* 1)-dimensional subspace] are defined by


$$\begin{array}{ll} V_{\lambda }=\text{span}\left\{\;\;\left(\;\;\begin{array}{c} \lambda \\ 1\\ \vdots \\ 1 \end{array}\;\;\right)\;\;\right\},\;V_{⊥}^{p_{l}}=\left\{\;\;\left(\;\;\;\begin{array}{c} 0\\ v_{c_{1}}\\ \vdots \\ v_{c_{\alpha }} \end{array}\;\;\;\right)\;\;:\;\overset{\alpha }{\underset{i=1}{\sum }}p_{l}^{c_{i}}v_{c_{i}}=0\;\right\}. & \; \end{array}$$ (17)

The full parametric gauge, denoted by $\Theta^{\lambda ,p}$, is defined to be the tensor product of these single-position gauges:


$$\begin{array}{ll} \Theta^{\lambda ,p}=\overset{L}{\underset{l=1}{⊗}}\Theta_{l}^{\lambda ,p}. & \; \end{array}$$ (18)

As detailed in S1 Text Sec 5, the corresponding projection matrix $P^{\lambda ,p}$ is found to have elements given by


$$\begin{array}{cc} P_{s^{\prime }t^{\prime }}^{\lambda ,p}=\underset{\begin{array}{l} l\;\text{s.t.}\\ {s^{\prime }}_{l}\in A\\ t_{l}^{\prime }\in A \end{array}}{\prod }\;\;\;\left(\delta_{s_{l}^{\prime }t_{l}^{\prime }}–p_{l}^{t_{l}^{\prime }}\eta \right)\;\times \;\;\;\underset{\begin{array}{c} l\;\text{s.t.}\\ s_{l}^{\prime }=*\\ t_{l}^{\prime }\in A \end{array}}{\prod }\;\;\;\left(p_{l}^{t_{l}^{\prime }}\eta \right)\;\times \;\;\;\underset{\begin{array}{c} l\;\text{s.t.}\\ s_{l}^{\prime }\in A\\ t_{l}^{\prime }=* \end{array}}{\prod }\;\;\;\left(1–\eta \right)\;\times \;\;\;\underset{\begin{array}{c} l\;\text{s.t.}\\ s_{l}^{\prime }=*\\ t_{l}^{\prime }=* \end{array}}{\prod }\;\;\eta , & \end{array}$$ (19)

where *η* = *λ*/(1 + *λ*) and where the augmented sequences $s^{\prime }$ and $t^{\prime }$ respectively index rows and columns. We thus obtain an explicit formula for the projection matrix needed to project any parameter vector into any gauge in the parametric family.

Gauges in the parametric family also have penalization matrices of a simple diagonal form. Specifically, if 0 < *λ* < *∞* and $p(s^{\prime })>0$ everywhere, Eq (10) is satisfied by the penalization matrix *Λ* having elements


$$\begin{array}{ll} \Lambda_{s^{\prime }t^{\prime }}=p(s^{\prime })\lambda^{o(s^{\prime })}\delta_{s^{\prime }t^{\prime }}, & \; \end{array}$$ (20)

where $o(s^{\prime })$ denotes the order of interaction described by $s^{\prime }$ (i.e., the number of non-star characters in $s^{\prime }$) and $p(s^{\prime })$ is defined as in Eq (15) but with $p_{l}^{s_{l}^{\prime }}=1$ when $s_{l}^{\prime }=*$. See S1 Text Sec 5 for a proof. Note that, although Eq (20) does not hold when *λ* = 0, when *λ* = *∞*, or when $p_{l}^{c}=0$ for any choice of *c* and *l*, one can still interpret $\Theta^{\lambda ,p}$ [which is well-defined in Eq (18) and Eq (19)] as arising from Eq (10) under a limiting series of penalization matrices *Λ*.

#### Trivial gauge.

Choosing *λ* = 0 yields what we call the “trivial gauge”. In the trivial gauge, $\theta_{s^{\prime }}=0$ if $s^{\prime }$ contains one or more star characters [by Eq (19)], and so the only nonzero parameters correspond to interactions of order *L*. As a result,


$$\begin{array}{ll} f_{\text{all}}(s,\vec{\theta })=\theta_{s} & \; \end{array}$$ (21)

for every sequence *s* ∈ *S*. Note in particular that the trivial gauge is unaffected by *p*. Thus, the trivial gauge essentially represents sequence-function relationships as catalogs of activity values, one value for every sequence. See S1 Text Sec 6 for details.

#### Euclidean gauge.

Choosing *λ* = *α* and choosing *p* to be the uniform distribution recovers what we call the “euclidean gauge”. In the euclidean gauge, the penalizing norm in Eq (10) is the standard euclidean norm, i.e.


$$\begin{array}{ll} ||\vec{\theta }|{|}_{\Lambda }^{2}=\underset{s^{\prime }}{\sum }\theta_{s^{\prime }}^{2}. & \; \end{array}$$ (22)

In S1 Text Sec 6 we show that the euclidean gauge is equal to the embedding space *E* and that parameter inference using standard $L_{2}$ regularization (i.e. choosing *Λ* to be a positive multiple of the identity matrix) will yield parameters in *E*.

#### Equitable gauge.

Choosing *λ* = 1 and letting *p* vary recovers what we call the “equitable gauge”. In the equitable gauge, the penalizing norm is


$$\begin{array}{cc} ||\vec{\theta }|{|}_{\Lambda }^{2}=\underset{s^{\prime }}{\sum }p(s^{\prime })\theta_{s^{\prime }}^{2}=\underset{s^{\prime }}{\sum }{\left\langle \;f_{s^{\prime }}^{\;2}\right\rangle }_{p}=\underset{s^{\prime }}{\sum }||\;f_{s^{\prime }}|{|}_{p}^{2}, & \end{array}$$ (23)

where $f_{s^{\prime }}=\theta_{s^{\prime }}x_{s^{\prime }}$ denotes the contribution to the activity landscape corresponding to the sequence feature $s^{\prime }$, ${\left\langle \cdot \right\rangle }_{p}$ denotes an average over sequences drawn from *p*, and $||\;f\;|{|}_{p}^{2}=\sum_{s\in S}p(s)f{\left(s\right)}^{2}$ is the squared norm of a function *f* on sequence space with respect to *p*. The equitable gauge thus penalizes each parameter $\theta_{s^{\prime }}$ in proportion to the fraction of sequences that parameter applies to. Equivalently, the equitable gauge can be thought of as minimizing the sum of the squared norms of the landscape contributions $||\;f_{s^{\prime }}|{|}_{p}^{2}$ rather than the squared norm of the parameter values themselves. Unlike the euclidean gauge, the equitable gauge accounts for the fact that different model parameters can affect vastly different numbers of sequences and can thereby have vastly different impacts on the activity landscape. See S1 Text Sec 6 for details.

#### Hierarchical gauge.

Choosing arbitrary *p* and taking *λ* → *∞* yields what we call the “hierarchical gauge”. When expressed in the hierarchical gauge, model parameters obey the marginalization property,


$$\begin{array}{ll} \underset{c_{k}}{\sum }p_{l_{k}}^{c_{k}}\theta_{l_{1}\ldots l_{K}}^{c_{1}\ldots c_{K}}=0, & \; \end{array}$$ (24)

for all interaction orders *K*, all choices of *K* positions $l_{1},\ldots ,l_{K}$, all choices of characters $c_{1},\ldots ,c_{K}$ at these positions, and all choices of index *k* = 1, …, *K*. This marginalization property has important consequences that we now summarize. See S1 Text Sec 7 for proofs of these results.

A first consequence of Eq (24) is that, when parameters are expressed in the hierarchical gauge, the mean activity among sequences matched by an augmented sequence can be expressed as a simple sum of parameters. For example,


$$\begin{array}{ccc} {\left\langle f_{all}\right\rangle }_{p} & = & \theta_{0}, \end{array}$$ (25)

$$\begin{array}{ccc} {\left\langle f_{all}\;|\;c\;at\;l\right\rangle }_{p} & = & \theta_{0}+\theta_{l}^{c}, \end{array}$$ (26)

$$\begin{array}{ccc} {\left\langle f_{all}\;|\;c\;at\;l,c^{\prime }\;at\;l^{\prime }\right\rangle }_{p} & = & \theta_{0}+\theta_{l}^{c}+\theta_{l^{\prime }}^{c^{\prime }}+\theta_{ll^{\prime }}^{cc^{\prime }}, \end{array}$$ (27)

and so on. Consequently, the parameters themselves can also be expressed in terms of differences of these average values. For instance, $\theta_{l}^{c}={\left\langle \;f_{all}\;|\;c\;at\;l\right\rangle }_{p}$ – ${\left\langle \;f_{all}\right\rangle }_{p}$. Because *p* factorizes by position, conditioning on having particular characters in a subset of positions is equivalent to the probability distribution produced by drawing sequences from *p* and then fixing those positions in the drawn sequences to those specific characters. Thus, $\theta_{l}^{c}$ can also be interpreted as the average effect of mutating position *l* to character *c* when sequences are drawn from *p*. Similarly, $\theta_{ll^{\prime }}^{cc^{\prime }}$ is the average effect, when drawing sequences from *p*, of fixing the character at position *l* to *c* and the one at $l^{\prime }$ to $c^{\prime }$ beyond what would be expected based on the effects of changing *l* to *c* and $l^{\prime }$ to $c^{\prime }$ individually (i.e., epistasis). Higher-order coeﬃcients have a similar interpretation. The hierarchical gauge thus provides an ANOVA-like decomposition of activity landscapes.

A second consequence of Eq (24) is that the activity landscape, when expressed in the hierarchical gauge, naturally decomposes into mutually orthogonal components. Let *σ* denote a set comprising all augmented sequences that have the same pattern of star and non-star positions, and let $f_{\sigma }=\sum_{s^{\prime }\in \sigma }\theta_{s^{\prime }}\;x_{s^{\prime }}$ be the corresponding component of $f_{all}$. These landscape components are *p*-orthogonal when expressed in the hierarchical gauge:


$$\begin{array}{ll} {\left\langle f_{\sigma }f_{\tau }\right\rangle }_{p}=\delta_{\sigma \tau }\underset{s^{\prime }\in \sigma }{\sum }p(s^{\prime })\;\theta_{s^{\prime }}^{2}, & \; \end{array}$$ (28)

where *σ* and *τ* represent any two such sets of augmented sequences. One implication of this orthogonality relation is that the variance of the landscape (with respect to *p*) is the sum of contributions from interactions of different orders:


$$\begin{array}{ll} {var}_{p}[\;f\;]=\overset{L}{\underset{k=0}{\sum }}{var}_{p}[\;f_{k}], & \; \end{array}$$ (29)

where $f_{k}$ denotes the sum of all *k*-order terms that contribute to $f_{all}$. Another implication is that the hierarchical gauge minimizes the variance attributable to different orders of interaction in a hierarchical manner: higher-order terms are prioritized for variance minimization over lower-order terms, and within a given order parameters are penalized in proportion to the fraction of sequences they apply to.

A third consequence of Eq (24) is that hierarchical gauges preserve the form of a large class of one-hot models that are equivalent to all-order interaction models with certain parameters fixed at zero (specifically, these models satisfy the condition that if a parameter for a sequence feature is fixed at zero, all higher-order sequence features contained within that sequence feature also have their parameters fixed at zero). These models, which we call the “hierarchical models,” include all-order interaction models in which the parameters above a specified order are zero (e.g., additive models and pairwise-interaction models), but also include other models, such as nearest-neighbor interaction models. Projecting onto the hierarchical gauge (but not other parametric family gauges) is guaranteed to produce a parameter vector where the appropriate entries are still fixed to be zero.

#### Zero-sum gauge.

The zero-sum gauge (illustrated in Figs 1A and 2A) is the hierarchical gauge for which *p* is the uniform distribution. The name of this gauge comes from the fact that, when *p* is uniform, Eq (24) becomes


$$\begin{array}{ll} \underset{c_{k}}{\sum }\theta_{l_{1}\ldots l_{K}}^{c_{1}\ldots c_{K}}=0. & \; \end{array}$$ (30)

Prior studies [12,15] have characterized the zero-sum gauge for the all-order interaction model. Our formulation of the hierarchical gauge extends those findings and generalizes them to gauges defined by non-uniformly weighted sums of parameters.

#### Wild-type and generalized wild-type gauges.

The wild-type gauge (illustrated in Figs 1B and 2B) is a hierarchical gauge that arises in the limit as *p* approaches an indicator function for some wild-type sequence, $s^{\text{wt}}$. In the wild-type gauge, only the parameters $\theta_{s^{\prime }}$ for which $s^{\prime }$ matches $s^{\text{wt}}$ receive any penalization, and all these penalized $\theta_{s^{\prime }}$ (except for $\theta_{0}$) are therefore driven to zero by minimization of the *Λ*-norm. Consequently, $\theta_{0}$ quantifies the activity of the wild-type sequence, each $\theta_{l}^{c}$ quantifies the effect of a single mutation to the wild-type sequence, each $\theta_{ll^{\prime }}^{cc^{\prime }}$ quantifies the epistatic effect of two mutations to the wild-type sequence, and so on. However, seeing the wild-type gauge as a special case of the hierarchical gauge provides the possibility of generalizing the wild-type gauge by using a *p* that is not the indicator function on a single sequence but rather defines a distribution over one or more alleles per position that can be considered as being “wild-type” (equivalently, the frequencies of some subset of position-specific characters are set to zero). Examples illustrating the utility of different choices for *p* are provided below. These gauges all inherit the property from the hierarchical gauge that their coeﬃcients relate to the average effect of taking draws from the probability distribution defined by *p* and setting a subset of positions to the characters specified by that coeﬃcient. More rigorously, these gauges are defined by considering the limit as $\epsilon \to 0^{+}$ of the hierarchical gauge with factorizable distribution


$$\begin{array}{ll} p_{\epsilon }(s)=\underset{l}{\prod }\left[(1–\epsilon )p_{l}^{s_{l}}+\frac{\epsilon }{\alpha }\right], & \; \end{array}$$ (31)

where the $p_{l}^{s_{l}}\geq 0$ are the position-specific factors of the desired nonnegative vector of probabilities *p*.

**Fig 3:**
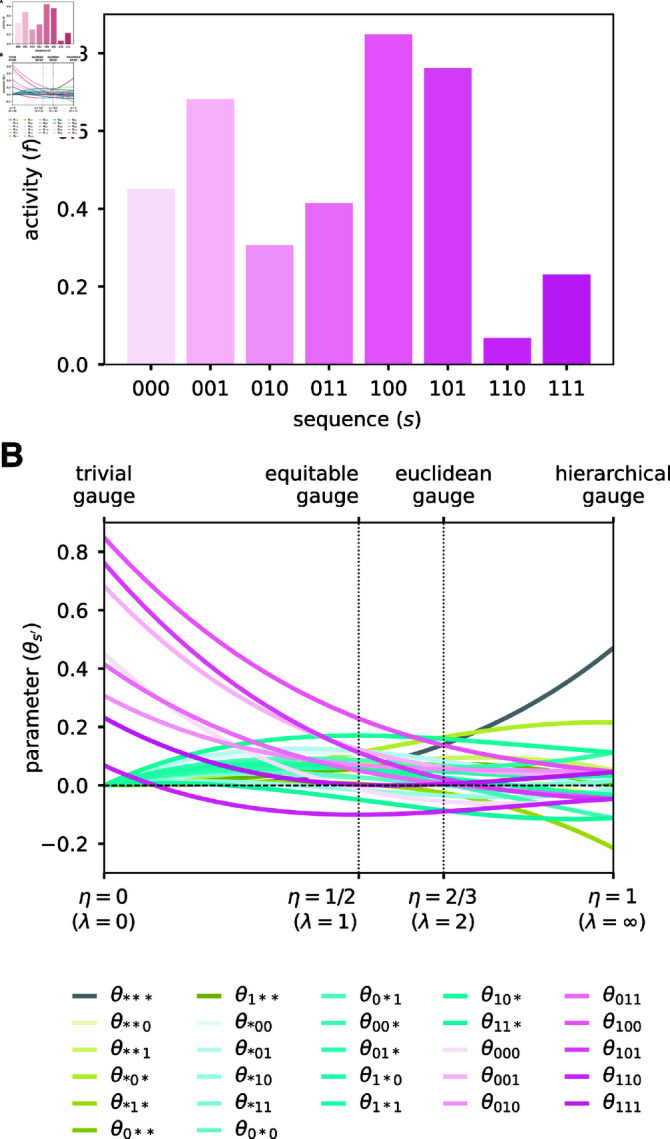
Binary landscape expressed in various parametric family gauges. (A) Simulated random activity landscape for binary sequences of length *L* = 3. (B) Parameters of the all-order interaction model for the binary landscape as functions of *η* = *λ*/(1 + *λ*). Values of *η* corresponding to different named gauges are indicated. Note: because the uniform distribution is assumed in all these gauges, the hierarchical gauge is also the zero-sum gauge.

### Applications

We now demonstrate the utility of our results on two example models of complex sequence-function relationships. First, we study how the parameters of the all-order interaction model behave under different parametric gauges in the context of a simulated landscape on short binary sequences. Although a number of studies have reported combinatorially complete landscapes in diverse biological systems (e.g., [46,47,59–65], focusing on this small simulated landscape allows us to better observe the nontrivial collective behavior that model parameters exhibit across different choices of gauge. Second, we examine the parameters of an empirical pairwise-interaction model for protein GB1 using the zero-sum and multiple generalized wild-type gauges. We observe how these different hierarchical gauges enable different interpretations of model parameters and facilitate the derivation of simplified models that are approximately correct in different localized regions of sequence space. The results provide intuition for the behavior of the various parametric gauges, and show in particular how hierarchical gauges can be used to explore and interpret real sequence-function relationships.

#### Gauge-fixing a simulated landscape on short binary sequences.

To illustrate the consequences of choosing gauges in the parametric family, we consider a simulated random landscape on short binary sequences. Consider sequences of length *L* = 3 built from the alphabet *A* = *{*0, 1*}*, and assume that the activities of these sequences are as shown in Fig 3A. The corresponding all-order interaction model has ${\left(\alpha \;+\;1\right)}^{L}=27$ parameters, which we index using augmented sequences: 1 constant parameter ($\theta_{***}$), 6 additive parameters ($\theta_{0**}$, $\theta_{1**}$, $\theta_{*0*}$, $\theta_{*1*}$, $\theta_{**0}$, $\theta_{**1}$), 12 pairwise parameters ($\theta_{00*}$, $\theta_{01*}$, $\theta_{10*}$, $\theta_{11*}$, $\theta_{0*0}$, $\theta_{0*1}$, $\theta_{1*0}$, $\theta_{1*1}$, $\theta_{*00}$, $\theta_{*01}$, $\theta_{*10}$, $\theta_{*11}$), and 8 third-order parameters ($\theta_{000}$, *θ*_001_, *θ*_010_, *θ*_011_, *θ*_100_, *θ*_101_, *θ*_110_, *θ*_111_).

We now consider what happens to the values of these 27 parameters when they are expressed in different parametric gauges, Θ^*λ*,*p*^. Specifically, we assume that *p* is the uniform distribution (though analogous results hold for other choices of *p*) and vary the parameter *λ* from 0 to *∞* (equivalently *η* varies from 0 to 1). Note that each entry in the projection matrix *P*^*λ*,*p*^ (Eq 19) is a cubic function of *η* due to *L* = 3. Consequently, each of the 27 gauge-fixed model parameters is a cubic function of *η* (Fig 3B). In the trivial gauge (*λ* = 0, *η* = 0), only the 8 third-order parameters are nonzero and the values of these parameters correspond to the values of the landscape at the 8 corresponding sequences. In the equitable gauge (*λ* = 1, *η* = 1/2), the spread of the 8 third-order parameters about zero is larger than that of the 12 pairwise parameters, which is larger than that of the 6 additive parameters, which is larger than that of the constant parameter. In the euclidean gauge (*λ* = 2, *η* = 2/3), the parameters of all orders exhibit a similar spread about zero. In the hierarchical gauge (*λ* = *∞*, *η* = 1), the spread of the 8 third-order parameters about zero is smaller than that of the 12 pairwise parameters, which is smaller than that of the 6 additive parameters, which is smaller than that of the constant parameter. Moreover, the marginalization and orthogonality properties of the hierarchical gauge fix certain parameters to be equal or opposite to each other, e.g., *θ*_1∗∗_ = −*θ*_0∗∗_ and the third order parameters are all equal up to their sign, which depends only on whether the corresponding sequence feature has an even or odd number of “1”s.

This example illustrates generic features of the parametric gauges. For any all-order interaction model on sequences of length *L*, the entries of the projection matrix *P*^*λ*,*p*^ will be *L*-order polynomials in *η*. Consequently, the values of model parameters, when expressed in the gauge Θ_*λ*,*p*_, will also be *L*-order polynomials in *η*. In the trivial gauge, only the highest-order parameters will be nonzero. In the equitable gauge, the spread about zero will tend to be smaller for lower-order parameters relative to higher-order parameters. In the euclidean gauge, parameters of all orders will exhibit similar spread about zero. In the zero-sum gauge, the spread about zero will tend to be minimized for higher-order parameters relative to lower-order parameters. The nontrivial quantitative behavior of model parameters in different parametric gauges thus underscores the importance of choosing a specific gauge before quantitatively interpreting parameter values.

**Fig 4:**
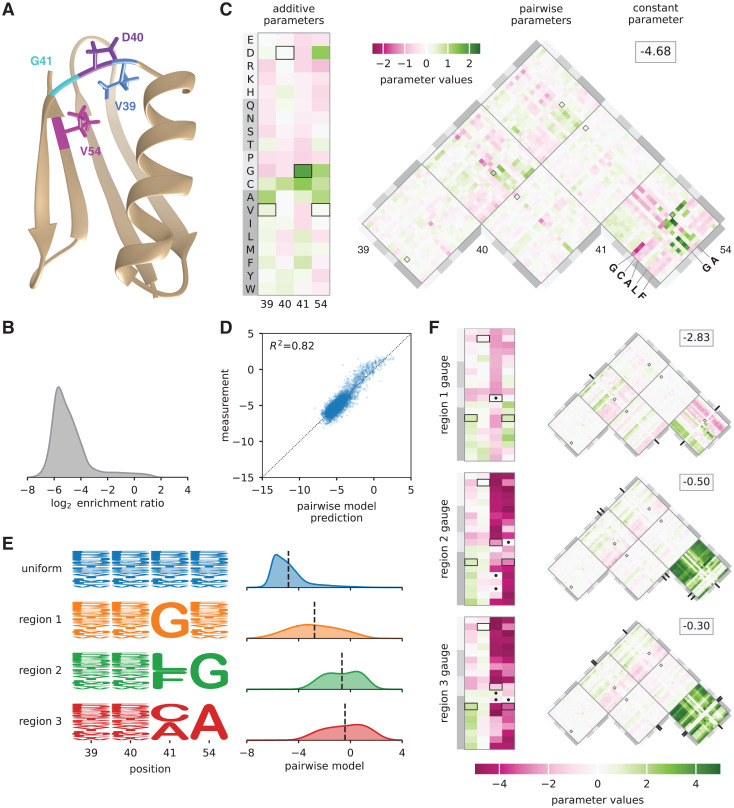
Landscape exploration using hierarchical gauges. (A) NMR structure of GB1, with residues V39, D40, G41, and V54 shown (PDB: 3GB1, from [66]). (B) Distribution of log_2_ enrichment relative to wild-type measured by [60] for nearly all 160,000 GB1 variants having mutations at positions 39, 40, 41, and 54. (C) Pairwise interaction model parameters inferred from the data of [60], expressed in the uniform hierarchical gauge (i.e., the zero-sum gauge). Boxes indicate parameters contributing to the wild-type sequence, VDGV. (D) Performance of pairwise-interaction model. Axes reflect log_2_ enrichment values relative to wild-type. Each dot represents a randomly chosen variant GB1 protein assayed by [60]. For clarity, only 5,000 of the ∼160,000 assayed GB1 variants are shown. (E) Probability logos [39] for uniform, region 1, region 2, and region 3 sequence distributions. Distributions of pairwise interaction model predictions for each region are also shown. (F) Model parameters expressed in the region 1, region 2, and region 3 hierarchical gauges. Dots and tick marks indicate region-specific constraints. Probability densities (panels B and D) were estimated using DEFT [45]. Pairwise interaction model parameters were inferred by least-squares regression using MAVE-NN [39]. Regions 1, 2, and 3 were defined based on [64]. NMR: nuclear magnetic resonance. GB1: domain B1 of protein G.

#### Hierarchical gauges of an empirical landscape for protein GB1.

Projecting model parameters onto different hierarchical gauges can facilitate the exploration and interpretation of sequence-function relationships. To demonstrate this application of gauge fixing, we consider an empirical sequence-function relationship describing the binding of the GB1 protein to immunoglobulin G (IgG). Wu et al. [60] performed a deep mutational scanning experiment that measured how nearly all 20^4^ = 160, 000 amino acid combinations at positions 39, 40, 41, and 54 of GB1 affect GB1 binding to IgG. These data report log_2_ enrichment values for each assayed sequence relative to the wild-type sequence at these positions, VDGV (Fig 4A and 4B). Using these data and least-squares regression, we inferred a pairwise-interaction model for log_2_ enrichment as a function of protein sequence at these *L* = 4 variable positions. The resulting model comprises 1 constant parameter, 80 additive parameters, and 2400 pairwise parameters (Fig 4C). While the model fits the data reasonably well (Fig 4D; *R*^2^ = 0.82), the deviation from measurements is still greater than that expected by experimental uncertainty and can be further reduced by using a more complex model (e.g., one that includes a global epistasis nonlinearity [9,67]). Nevertheless, the pairwise-interaction model serves well to illuminate the utility of different gauge-fixing strategies. To understand the structure of the activity landscape described by the pairwise interaction model, we now examine the values of model parameters in multiple hierarchical gauges. Explicit formulae for implementing hierarchical gauges for pairwise-interaction models are given in S1 Text Sec 8.

Fig 4C shows the parameters of the pairwise interaction model expressed in the hierarchical gauge corresponding to a uniform probability distribution on sequence space (i.e., the zero-sum gauge). In the zero-sum gauge, the constant parameter *θ*_0_ equals the average activity of all sequences. We observe *θ*_0_ = −4.68, indicating that a typical random sequence is depleted approximately 20-fold relative to the wild-type sequence, which the pairwise interaction model assigns a score of *–.*21. This finding confirms the expectation that a random sequence should be substantially less functional than the wild-type sequence.

The additive parameters in the zero-sum gauge are shown in the rectangular heat map in Fig 4C, and each additive parameter is equal to the difference between the mean activity of the set of sequences containing the corresponding amino acid at the relevant position relative to the mean activity of random sequences. We observe that the wild-type sequence receives positive or near-zero contributions at every position, including a contribution from the most positive additive parameter, corresponding to G at position 41. The additive parameters at positions 39, 40, and 54 that contribute to the wild-type sequence, however, are not the largest additive parameters at these positions. Moreover, the additive parameters that contribute to the wild-type sequence only sum to 2.32, meaning that even in the zero-sum gauge (which minimizes the variance due to pairwise parameters), of the total difference (4.47) between the wild-type score and the average sequence score, almost half (2.15) is due to contributions from pairwise parameters.

The pairwise parameters in the zero-sum gauge are shown in the triangular heat map in Fig 4C. Here, each pairwise parameter is equal to the difference between (i) the observed mean of the sequences containing the specified pair of characters at the specified pair of positions, and (ii) the expected mean activity based on the mean activity of sequences containing the individual characters at those positions together with the grand mean activity. We observe that the three largest-magnitude pairwise contributions to the wildtype sequence are from the pair G41V54 (1.25), V39G41 (0.91), and D40G41 (-0.44), indicating that position 41 is a major hub of epistatic interactions contributing to the wild-type sequence. Moving to the landscape as a whole, we observe that the largest magnitude pairwise interactions link positions 41 and 54. Moreover, the strongest positive pairwise contributions are obtained when a small amino acid (G or A) is present at position 54, and a G, C, A, L, or P is present at position 41 (see also [49]). This finding provides insight into the chemical nature of the epistatic interactions that facilitate wild-type GB1 binding to IgG.

Previous work [64,68] identified three disjoint regions of sequence space (region 1, region 2, and region 3) that contain high-activity sequences as judged by the GB1 measurements of Wu et al. [60]. Region 1 comprises sequences with G at position 41; region 2 comprises sequences with L or F at position 41 and G at position 54; and region 3 comprises sequences with C or A at position 41 and A at position 54. To investigate the structure of the GB1 landscape within these three regions, we defined probability distributions that were uniform in each region of sequence space and zero outside (Fig 4E; see S1 Text Sec 8 for formal definitions of these regions). We then examined the values of the parameters of the pairwise-interaction model, with the parameters expressed in the hierarchical gauges corresponding to the probability distribution *p*(*s*) for each of the three regions (the “region 1 hierarchical gauge”, “region 2 hierarchical gauge”, and “region 3 hierarchical gauge”). Since some characters at positions 41 and 54 have their frequencies set to zero, these hierarchical gauges are in fact generalized wild-type gauges, and the additive and pairwise parameters can be interpreted in terms of the mean effects of introducing mutations to these specific regions of sequences space.

In the region 1 hierarchical gauge (Fig 4F, top), the additive parameters for position 41 quantify the effect of mutations away from G, and the additive parameters for positions 39, 40, and 54 quantify the average effect of mutations conditional on G at position 41. From the additive parameters at position 54, we observe that cysteine (C) and hydrophobic residues (A, V, I, L, M, or F) increase binding, and that proline (P) and charged residues (E, D, R, K) decrease binding. From the additive parameters at position 40, we observe that amino acids with a 5-carbon or 6-carbon ring (H, F, Y, W) increase binding, suggesting the presence of structural constraints on side chain shape, rather than constraints on hydrophobicity or charge. The largest pairwise parameters all involve mutations from G at position 41 to another amino acid, and careful inspection of these pairwise parameters show that they are roughly equal and opposite to the additive effects of mutations at the other three positions. This indicates a classical form of masking epistasis, where the typical effect of a mutation at position 41 results in a more or less complete loss of function, after which mutations at the remaining three positions no longer have a substantial effect.

In the region 2 hierarchical gauge (Fig 4F, middle), the additive parameters at position 54 quantify the average effect of mutations away from G contingent on L or F at position 41, the additive parameters at position 41 quantify the average effects of mutations away from L or F contingent on G at position 54, and the additive parameters at positions 39 and 40 quantify the average effects of mutations contingent on L or F at position 41 and on G at position 54. From the values of the additive parameters, we observe that mutations away from L or F at position 41 in the presence of G at position 54 are typically strongly deleterious (mean effect –3.39), and that mutations away from G at position 54 in the presence of L or F at position 41 are also strongly deleterious (mean effect –3.75). However, the pairwise parameters linking positions 41 and 54 are strongly positive (mean effect 2.85), again indicating a masking effect where the first deleterious mutation at position 41 or 54 results in a more or less complete loss of function, so that an additional mutation at the other position has little effect. Note also the similar but less extreme pattern of masking between the large effect mutations at positions 41 and 54 with the milder mutations at positions 40 and 41, whose interaction coeﬃcients are of the opposite sign of the additive effects at positions 40 and 41. Similar results hold for the region 3 hierarchical gauge, where mutations at positions 41 and 54 have masking effects on each other as well as on mutations in the other two positions (Fig 4F, bottom). However, we can also contrast patterns of mutational effects between these regions. For example, mutating position 54 to G (a mutation leading towards region 2) on average has little effect in region 1 but would be deleterious in region 3. Similarly, if we consider mutations leading from region 2 to region 3, we can see that mutating 41 to C in region 2 typically has little effect whereas mutating 41 to A is more deleterious.

**Fig 5:**
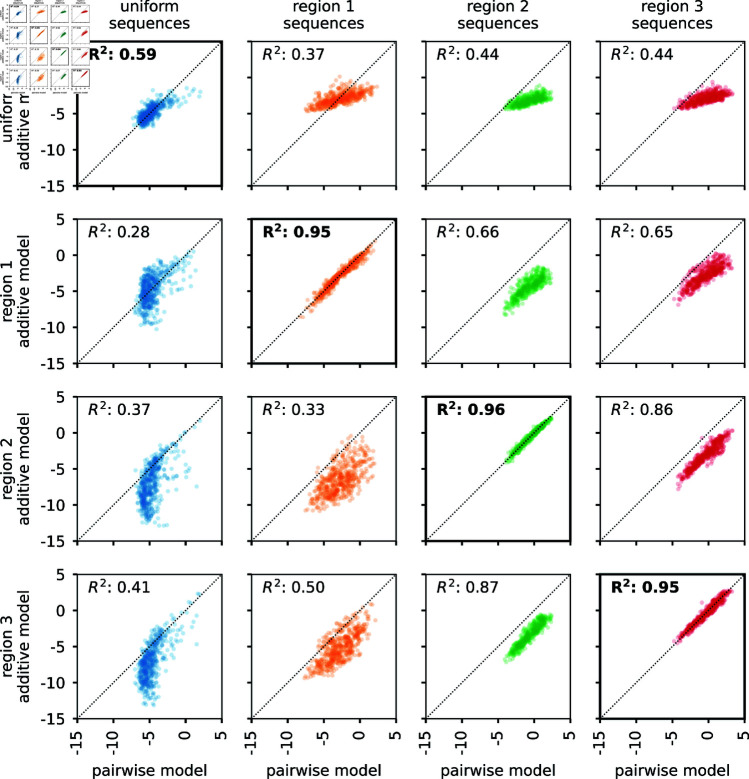
Model coarse-graining using hierarchical gauges. Shown are data for 500 random 4 aa sequences generated using each of the four distributions listed in Fig 4E (i.e., uniform, region 1, region 2, and region 3). Vertical axes show log_2_ enrichment (relative to wild-type) as predicted by additive models of GB1 derived by model truncation using region-specific zero-sum gauges (from Fig 4C and 4F). Horizontal axes show predictions of the full pairwise-interaction model. Diagonals indicate equality. GB1: domain B1 of protein G.

Besides using the interpretation of hierarchical gauge parameters as average effects of mutations to understand how mutational effects differ in different regions of sequence space, we hypothesized that by applying different hierarchical gauges to the pairwise interaction model, one might be able to obtain simple additive models that are accurate in different regions of sequence space. Our hypothesis was motivated by the fact that the parameters of all-order interaction models in the zero-sum gauge are chosen to maximize the fraction of variance in the sequence-function relationship that is explained by lower-order parameters. To test our hypothesis, we defined an additive model for each of the four hierarchical gauges described above (uniform, region 1, region 2, and region 3) by projecting pairwise interaction model parameters onto the hierarchical gauge for that region, then setting all the pairwise parameters to zero. We then evaluated the predictions of each additive model on sequences randomly drawn from each of the four corresponding probability distributions (uniform, region 1, region 2, and region 3). The results (Fig 5) show that the activities of sequences sampled uniformly from sequence space are best explained by the additive model derived from the zero-sum gauge, that the activities of region 1 sequences are best explained by the additive model derived from the region 1 hierarchical gauge, and so on for regions 2 and 3. In particular, additive models derived using region-specific gauges are far more accurate in their respective regions than is the additive model derived using the uniform (i.e., zero-sum) gauge. This shows that projecting a pairwise interaction model (or other hierarchical one-hot model) onto the hierarchical gauge corresponding to a specific region of sequence space can sometimes be used to obtain simplified models that approximate predictions by the original model in that region.

## Discussion

Here we report a unified strategy for fixing the gauge of commonly used models of sequence-function relationships. First we defined a family of analytically tractable gauges for the all-order interaction model. We then derived explicit formulae for imposing any of these gauges on model parameters, and used these formulae to investigate the mathematical properties of these gauges. The results show that these linear gauges include all of the most commonly used gauges in the literature (even though most possible gauges, both linear and nonlinear, are not members of this family). We also find that a subset of these gauges (the hierarchical gauges) can be applied to diverse lower-order models including additive models, pairwise-interaction models, and higher-order interaction models.

Next, we demonstrated the family of gauges in two contexts: a simulated all-order interaction landscape on short binary sequences, and an empirical pairwise-interaction landscape for the protein GB1. The GB1 results, in particular, show how applying different hierarchical gauges can facilitate the biological interpretation of complex models of sequence-function relationships and the derivation of simplified models that are approximately correct in localized regions of sequence space.

Our study was limited to linear models of sequence-function relationships. Although linear models are used in many computational biology applications, more complex models are becoming increasingly common. For example, linear-nonlinear models (which include global epistasis models [9,67,69,70] and thermodynamic models [37,39,71–74]) are commonly used to describe fitness landscapes and/or sequence-dependent biochemical activities. The gauge-fixing strategies described here remain applicable to the linear part of linear-nonlinear models. We note, however, that such models often have additional gauge freedoms, such as diffeomorphic modes [75,76], that also need to be fixed before parameter values can be meaningfully interpreted.

Sloppy modes are another important issue to address when interpreting quantitative models of sequence-function relationships. Sloppy modes are directions in parameter space that (unlike gauge freedoms) do affect model predictions but are nevertheless poorly constrained by data [77,78]. Understanding the mathematical structure of sloppy modes, and developing systematic methods for fixing these modes, is likely to be more challenging than understanding gauge freedoms. This is because sloppy modes arise from a confluence of multiple factors: the mathematical structure of a model, the distribution of data in feature space, and measurement uncertainty. Nevertheless, understanding sloppy modes is likely to be as important in many applications as understanding gauge freedoms. We believe the study of sloppy modes in quantitative models of sequence-function relationships is an important direction for future research.

Deep neural network (DNN) models present perhaps the biggest challenge for parameter interpretation. DNN models have had remarkable success in quantitatively modeling biological sequence-function relationships, most notably in the context of protein structure prediction [79,80], but also in the context of other processes including transcriptional regulation [81–83], epigenetics [84–86], and mRNA splicing [87,88]. It remains unclear, however, how researchers might gain insights into the molecular mechanisms of biological processes from inferred DNN models. DNNs are by nature highly over-parameterized [89–91], making the direct interpretation of DNN parameters infeasible. Instead, a variety of attribution methods have been developed to facilitate DNN model interpretations [92–95]. Existing attribution methods can often be thought of as providing additive models that approximate DNN models in localized regions of sequence space [96], and the presence of gauge freedoms in these additive models needs to be addressed when interpreting attribution method output (as in [40,97]). We anticipate that, as DNN models become more widely adopted for mechanistic studies in biology, there will be a growing need for attribution methods that provide more complex quantitative models that approximate DNN models in localized regions of sequence space [16]. If so, a comprehensive mathematical understanding of gauge freedoms in parametric models of sequence-function relationships will be needed to aid in these DNN model interpretations.

## Supporting information

S1 Text Supporting information text. (PDF)
